# Hypodipsic-hypernatremia syndrome in an adult with polycythemia: a case report

**DOI:** 10.1186/s13256-018-1938-y

**Published:** 2018-12-27

**Authors:** Mogamat-Yazied Chothia, Kiran George, Muhammed Sheik, Mogamat Razeen Davids

**Affiliations:** 1 Division of Nephrology, Department of Medicine, Tygerberg Hospital and Stellenbosch University, Ward A7, Tygerberg Hospital, Francie van Zijl Drive, Parow Valley, Cape Town, 7505 South Africa; 2 Division of General Medicine, Department of Medicine, Tygerberg Hospital and Stellenbosch University, Cape Town, South Africa

**Keywords:** Idiopathic, Hypodipsia, Hypernatremia, Polycythemia

## Abstract

**Background:**

Hypernatremia is a very common electrolyte disorder and is frequently encountered in out-patient as well as in-hospital settings. We describe an adult who was found to have unexplained relative polycythemia and episodic hypernatremia. A diagnosis of idiopathic hypodipsic-hypernatremia syndrome was made and the patient was managed with a water-drinking schedule.

**Case presentation:**

A 24-year-old South African-Indian man was found to have polycythemia in association with episodes of hypernatremia. Investigations indicated that he had relative polycythemia. He experienced no thirst at a time when his serum sodium concentration was found to be 151 mmol/L. Further testing indicated that his renal response to arginine vasopressin was intact and magnetic resonance imaging of his brain revealed no hypothalamic lesions. A diagnosis of idiopathic hypodipsic-hypernatremia syndrome was made and he was managed with a water-drinking schedule that corrected his hypernatremia.

**Conclusion:**

Hypodipsia should always be considered when a patient without physical or cognitive disability presents with unexplained episodic hypernatremia or with relative polycythemia.

## Background

Hypernatremia (serum sodium concentration >  145 mmol/L) is a very common electrolyte disorder and is frequently encountered in out-patient as well as in-hospital settings [1, 2]. Since the main determinant of serum osmolality is the serum sodium concentration, changes in the concentration of the latter reflect changes in serum osmolality. Osmoreceptors in the hypothalamus respond to changes in serum osmolality by stimulating or inhibiting the sensation of thirst, as well as by controlling the release of arginine vasopressin (AVP) from the neurohypophysis. The latter is a critical element in the renal conservation or excretion of water. Thus, hypernatremia indicates dysfunction of the control mechanisms for serum osmolality. In those without physical and/or cognitive disabilities, the presence of chronic hypernatremia invariably indicates an inability to sense thirst. In fact, studies conducted in patients with diabetes insipidus have found that the severity of hypernatremia was dependent on the ability to sense thirst [3]. Isolated hypodipsia with an intact AVP response is a very rare phenomenon. It may be caused by congenital or acquired disease affecting the hypothalamic area and includes conditions such as vascular malformations, malignancies, trauma, and granulomatous disease [4, 5].

We describe an adult who was found to have unexplained relative polycythemia and episodic hypernatremia. Following numerous investigations, it became clear that the patient was not able to sense thirst whereas the renal response to AVP was intact. A diagnosis of idiopathic hypodipsic-hypernatremia syndrome was made and the patient was managed with a water-drinking schedule. To the best of our knowledge, this case represents a first description of hypodipsic-hypernatremia syndrome presenting with unexplained relative polycythemia.

## Case presentation

A 24-year-old South African-Indian man, a medical student, had presented 6 years earlier to his peripheral hospital with complaints of lethargy and weakness, chronic constipation and lower backache, and episodes of hematochezia, associated with anorectal pain. These symptoms started 18 months earlier. The clinical examination was unremarkable. Routine blood tests were otherwise normal except for marked polycythemia (Table 1). A colonoscopy was performed, and a biopsy taken from a sessile rectal polyp; however, no specific pathological diagnosis could be made, and he was referred to our center for further management.

**Table 1:** The patient’s serum and urine biochemistry results

	Ref range	Aug 2012	Nov 2015	Jan 2016	Mar 2016	May 2016	Jun 2016	Feb 2017	Jul 2018	Aug 2018^a^	Sep 2018^b^
Serum											
Sodium	138–145 mmol/L	154	147	144	144	143	142	143	147	151	141
Potassium	3.3–5.3 mmol/L	5.1	5	4.6	4.9	4.8	5	4.4	4.6	5.5	
Urea	2.1–7.1 mmol/L	6.4	5.5	5	3.8	4.1	5.8	4.6	5.9	3.9	4.9
Creatinine	64–104 umol/L		107	94	101	93	89	106	110	99	92
Unadjusted calcium	2.15–2.50 mmol/L						2.52		2.56	2.72	2.43
Ionized calcium	1.1–1.3 mmol/L								1.21		
Leukocyte count	4.0–10.0 × 10^9^/L	8.6	10.8		9.0	8.0	8.2	10.1	12.9	8.9	8.8
Hemoglobin	13.0–17.0 g/dL	17.8	19.3		19.1	18.5	18.8	18.6	20.2	19.0	18.1
Hematocrit	0.40–0.50	0.55	0.60		0.60	0.56	0.58	0.58	0.69	0.63	0.58
Mean cell volume	79.1–98.9 fL	84.9	81.1		85.1	84	85.4	85.8	85.9	86.1	84.1
Platelets	137–373 × 10^9^/L	279	305		227	215	234	287	313	301	280
Albumin	35–52 g/L	52							52		
Erythropoietin	2.6–18.5 U/L		10.7								
25-OH-vitamin D	> 72.5 nmol/L					< 10.5					
PTH	2–7 pmol/L									3.6	
Renin	2–24 ng/L									45.6	
Aldosterone	50–640 pmol/L									176	
Cortisol	130–530 nmol/L									130	
Urine											
Sodium	mmol/L			174							
Osmolality	50–1200 mOsm/kg.H_2_O			1040			792				763

*Abbreviations*: *Unadj.calcium* unadjusted calcium, *25-OH vitamin D* 25-hydroxy vitamin D, *PTH* parathyroid hormone

^a^Date of nephrology consultation

^b^Nephrology consultation following 1 week of ~ 2 litres per day of water drinking

At our gastroenterology clinic, the initial presentation together with a review of the rectal biopsy were considered suggestive of ulcerative colitis and he was initiated on 5-aminosalicylic acid and, later, sulfasalazine. However, the symptoms did not improve, and these drugs were stopped. During this time, it was noticed that his blood pressure was 156/86 mmHg, and treatment with enalapril was initiated. After a period of approximately 4 months, the enalapril was stopped because he had no evidence of left ventricular hypertrophy on an electrocardiogram or echocardiography and 24-hour ambulatory blood pressure monitoring was normal. A repeat colonoscopy was performed, this time revealing rectal prolapse. The gastrointestinal symptoms resolved completely without the need for further medication.

During this period, he was also investigated for the polycythemia at our hematology clinic. A blood test for the Janus kinase-2 (V617F) mutation was negative and the blood erythropoietin concentration was normal (Table 1), hence, excluding primary polycythemia. Due to the severity of the polycythemia, he received frequent venesections that resulted in lethargy, and the venesections were subsequently stopped. Other investigations for true polycythemia were normal and included: a venous blood gas to determine the P50 value for hemoglobin oxygen affinity; echocardiography to rule out congenital cyanotic heart disease; polysomnography for obstructive sleep apnea syndrome; and an abdominal ultrasound for hepatocellular carcinoma, polycystic kidney disease, renal cell carcinoma, and renal arterial stenosis. A radioisotope study showed that the red cell mass was within the normal range; however, the plasma volume was reduced, suggesting relative polycythemia. After approximately 5 years, he was referred to the nephrology unit for further investigation.

At this time, there were no symptoms of nausea, vomiting, diarrhea, polyuria, or nocturia. Once again, he confirmed that he never felt thirsty, even during the previous episodes of hypernatremia. He drank water out of habit rather than in response to thirst. He was not using any prescription drugs, including diuretics. His family history included a father with diabetes mellitus and hypercholesterolemia and a mother with hypertension. He did not smoke tobacco or consume alcohol. He led a relatively sedentary lifestyle and did not participate in any outdoor recreational activities. He was in his final year of medical school training. A clinical examination was unremarkable. He was euvolemic and there was no evidence of postural hypotension. No cognitive or focal neurological deficits were identified. A urine dipstick was normal. His weight was 96 kg and height 1.70 m (calculated body mass index of 33.2 kg/m^2^). Bioimpedance spectroscopy (Body Composition Monitor ®, Fresenius Medical Care, Germany) was used to further assess his hydration status and revealed a total body water volume which was 800 mL less than predicted.

**Fig. 1:**
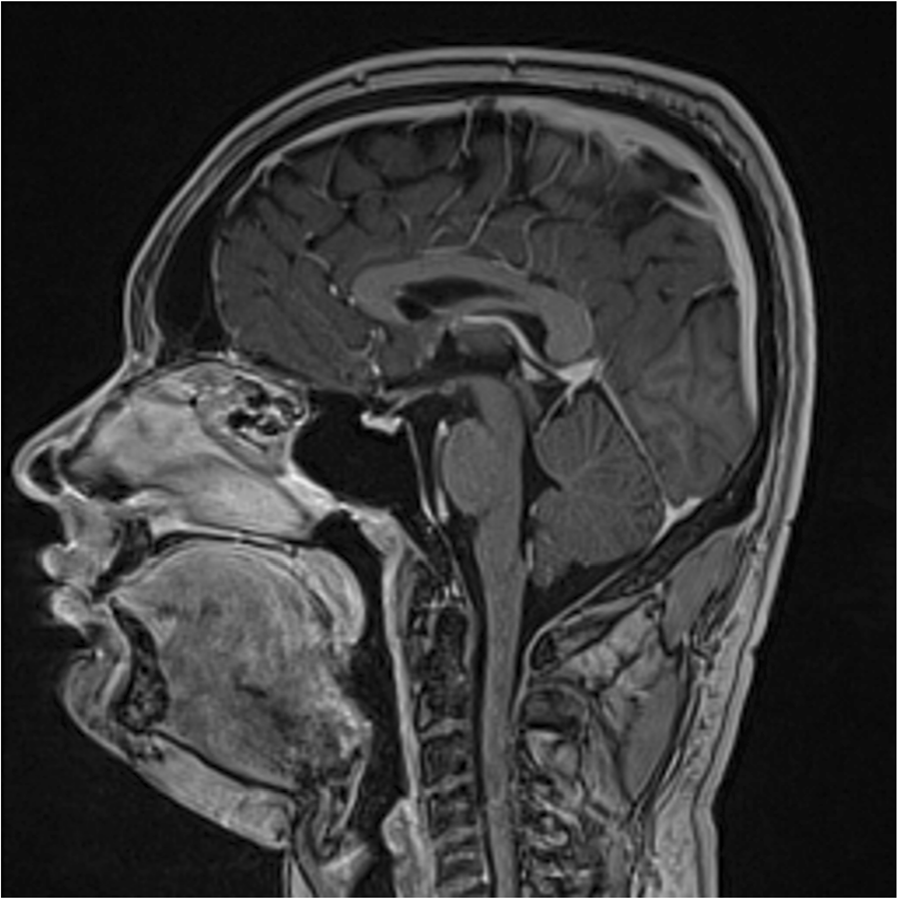
Magnetic resonance image of the brain (sagittal view) showing the absence of a hypothalamic lesion

A blood sample for serum sodium concentration was drawn and revealed a value of 151 mmol/L (reference range 138–145 mmol/L; see Table 1). Previous urine osmolality samples showed excellent urinary concentrating ability, thereby excluding a diagnosis of diabetes insipidus. A diagnosis of hypodipsia was made, and he was asked to drink 2 liters of water daily for 1 week. Blood and urine samples were then repeated and revealed normalization of the serum sodium concentration while maintaining urinary concentrating ability, thus confirming our suspicion of hypodipsia (Table 1). Since hypodipsia has been reported to be the result of a hypothalamic lesion, magnetic resonance imaging of his brain was performed but no intracranial pathology was identified (Fig. 1). He was instructed to continue drinking at least 2 liters of water daily. On follow-up 6 weeks later, he remained clinically well and did not report any significant change in his symptoms.

## Discussion

We present a challenging case of episodic hypernatremia and associated relative polycythemia that after a protracted period and following numerous investigations was found to have idiopathic hypodipsia. Frequently causes of relative polycythemia include diuretics, vomiting, and diarrhea [6]. To the best of our knowledge, this is the first description of hypodipsic-hypernatremia syndrome presenting with relative polycythemia.

Hypodipsic-hypernatremia syndrome is a rare disorder of reduced or absent thirst sensation. It frequently presents with recurrent episodes of hypernatremia in children or adults and may be caused by congenital or acquired neurological disorders. To the best of our knowledge, this is the first description of an adult who presented with unexplained polycythemia.

Hypernatremia may result from a gain of sodium or a loss of water. The former frequently occurs in critically ill, hospitalized patients who receive excessive amounts of intravenously administered isotonic fluids, while the latter occurs in the out-patient setting, usually due to conditions such as diarrhea. Regardless of the underlying mechanism, the presence of hypernatremia indicates dysfunction of the normal control mechanisms for water balance. A group of specialized hypothalamic cells function as osmoreceptors and, during hypernatremia, shrinks because of the efflux of water. This triggers the release of neuronal signals to the thirst center and the posterior pituitary gland [7]. Stimulation of the thirst center leads to increased water intake, and the release of AVP causes renal water retention and the excretion of a small volume of maximally concentrated urine [7].

Our patient was not critically ill and did not have a condition that caused excessive gastrointestinal or renal water losses, or which prevented him from gaining access to water. Therefore, a search for dysfunction of the water control axis was undertaken.

Following numerous investigations that eliminated causes of true polycythemia, the presence of reduced total body water, and hence relative polycythemia, was confirmed using radioisotope-labeled red blood cells and albumin-labeled plasma, as well as bioimpedance spectroscopy. Additional evidence that supported hypovolemia included a raised plasma renin concentration and a serum albumin concentration that was at the upper limit of normal. An interesting finding was that of hypercalcemia. This seemed to mirror the changes in serum sodium concentration. Since our laboratory no longer reports adjusted calcium concentrations, the hypercalcemia was thought to be the result of hyperalbuminemia due to hemoconcentration [8]. This was supported by the normal serum ionized calcium concentration, as well as a normal parathyroid hormone concentration.

AVP is primarily controlled by changes in serum osmolality; however, non-osmotic stimuli may also have an influence on its secretion, albeit to a lesser extent [9]. Cases of hypodipsia have been described where there appeared to be an intact urinary concentrating ability, but with AVP being secreted in response to the non-osmotic stimulus of hypovolemia. However, correction of the hypovolemia resulted in the unmasking of diabetes insipidus [10]. In our patient, the suspicion of isolated hypodipsia was heightened after investigations confirmed that urinary concentrating ability remained intact even after he increased his water intake.

A diagnosis of hypodipsia can readily be made in a patient that denies the sensation of thirst and/or does not spontaneously drink water when the serum sodium concentration is greater than 150 mmol/L or the serum osmolality is greater than 310 mOsm/kg [5]. In our patient, 4 out of 10 serum sodium concentrations were greater than 145 mmol/L. The normal serum sodium concentrations were probably due to the timing of the blood samples relative to our patient’s water consumption; he reported drinking water out of habit rather than in response to thirst. The diagnosis of hypodipsia can be confirmed by a water deprivation test, which documents the absence of thirst once the serum sodium concentration exceeds 150 mmol/L. In our patient, the serum sodium concentration was found to be 151 mmol/L at a time when he denied thirst. A formal water deprivation test was therefore not necessary.

Four categories of hypodipsia have been described [11]. In type A, a higher than normal serum osmolality is needed to stimulate thirst and vasopressin release; type B results in inadequate thirst stimulation and vasopressin release and may be caused by conditions that result in partial loss of osmoreceptors; type C is caused by complete loss of osmoreceptors and therefore complete absence of thirst and vasopressin release. This type frequently occurs following surgery. Our patient was thought to have type D hypodipsia. This rare form presents with isolated hypodipsia but with a normal response to vasopressin. A search of the literature identified only 14 cases and these, together with our case, are summarized in Table 2 [4, 5, 12–23]. The ages ranged from 5 to 56 years, 11 of the 15 patients were male, serum sodium concentrations ranged from 142 mmol/L to 194 mmol/L, and urine osmolality ranged from 623 to 1295 mOsm/kgH_2_O. Most cases (73%) had an identifiable cause and included psychiatric conditions (*n* = 3), brain tumors (2), viral infections (2), stroke (2), head trauma (1), and congenital hydrocephalus (1). In four cases, including the current case, no identifiable cause could be found. In those where AVP was measured, a normal response to changes in serum osmolality was reported. Of note, none of the other cases reported polycythemia.

**Table 2:** Reported cases of hypodipsic-hypernatremia with intact arginine vasopressin response

Case	Age at diagnosis (years)	Sex	Serum sodium concentration (mmol/L)	Urine osmolality (mOsm/kg)	Hb (g/dL) / Hct (%)	AVP concentration (pg/ml)	Final diagnosis	Treatment
Conley *et al*., 1976 [20]	7	F	194	1129	NR	ND	Idiopathic	Water-drinking schedule
Powell *et al*., 1983 [12]	9	M	145–165	> 1000	−/44.6	ND	Possible post-viral hypothalamic dysfunction	Diuretics Salt restriction Pitressin (vasopressin) Water-drinking schedule
Farley *et al*., 1986 [13]	17	M	181	645	NR	Normal response to saline infusion	Schizophrenia	Treatment of psychosis
Hammond *et al*., 1986 [5]	5	NR	150	1112–1138	Reported as normal	14.2–17.3 (free water-drinking and serum sodium concentration ranging from 149 to 150 mmol/L)	Congenital CMV	Water-drinking schedule
Assadi *et al*., 1989 [14]	6	F	187	854	14.5/43.9	25.6 (water deprivation test)	Head injury	Water-drinking schedule
Franco-Saenz *et al*., 1989 [23]	22	M	181	1295	−/42.8	3.3 pmol/L (normal AVP response: > 3.2 pmol/L when serum osmolality > 302 mOsm/kg)	Hydrocephalus	Intravenous fluids and forced water-drinking
Phillips and Gabow, 1990 [4]	34	M	165	898	NR	ND	Psychotic depression	Electroconvulsive therapy
Zünd *et al*., 1998 [22]	45	M	171	700	NR	3-fold rise in response to thirst test	Stroke	NR
Kang *et al*., 2001 [15]	25	M	168	623	11.9/35.3	> 14.16 (1.0–6.7) (water deprivation test)	Brain tumor	Radiotherapy, steroids, thyroxine, water-drinking schedule
López-Capapé *et al*., 2004 [16]	12	M	162	1230	NR	12.8 (2.5% salt load)	Idiopathic	Water-drinking schedule
Ramthun *et al*., 2011 [21]	56	M	157	1.026 (SG)	NR	NR	Hemorrhagic stroke	Water-drinking schedule
Mattai, 2011 [17]	36	F	176	1172	NR	NR	Craniopharyngioma or germ cell tumor	Initial treatment with 0.45% saline followed by oral intake
Sabzghabaei and Rastegar, 2015 [18]	27	M	171	840	NR	ND	Idiopathic	Water-drinking schedule
Manning *et al*., 2017 [19]	56	M	181	1080	NR	ND	Psychogenic adipsia	Water-drinking schedule Mirtazapine Clonazepam
Chothia; this case report	25	M	142–154	763–1040	17.8–20.2 / 55–69	ND	Idiopathic	Water-drinking schedule

*AVP* arginine vasopressin, *CMV* cytomegalovirus, *F* female, *Hb* hemoglobin, *Hct* hematocrit, *M* Male, *ND* not done, *NR* not reported, *SG* specific gravity

Management involves the prescription of a water-drinking schedule. This may be guided by the patient’s weight. Water consumption should be increased during warmer months and during exercise. Since the AVP response to serum osmolality is intact, patients will have a water diuresis in response to excessive intake and there is therefore no risk of water overload and cerebral edema.

Hypodipsic-hypernatremia syndrome seems to have an overall favorable outcome regardless of the underlying cause. In the cases cited in Table 2, there were no deaths reported. In most of the patients, the hypodipsia persisted and was treated with a water-drinking schedule only. Interestingly, in all cases that had an underlying psychiatric diagnosis, the hypodipsia resolved completely following treatment of the underlying condition.

## Conclusion

To the best of our knowledge, this is the first description of hypodipsia-hypernatremia syndrome presenting with unexplained relative polycythemia. Hypodipsia should always be considered when a patient without physical or cognitive disability presents with unexplained episodic hypernatremia or with relative polycythemia.

## Abbreviation

AVP: Arginine vasopressin; Unadj.calcium, unadjusted calcium; 25-OH vitamin D, 25-hydroxy vitamin D; PTH, parathyroid hormone.
